# OpenTriIDS: Confidence-Guided Open-Set Intrusion Triage for Unknown Attack Detection in IoT Devices

**DOI:** 10.3390/s26206369

**Published:** 2026-10-09

**Authors:** Xuan Wu, Peng Wang, Yafei Song, Xiaodan Wang

**Affiliations:** 1 College of Air and Missile Defense, Air Force Engineering University, Xi’an 710051, China; xuanwud@163.com (X.W.); yafei_song@163.com (Y.S.); 2 Academy of Military Science, Beijing 100091, China

**Keywords:** IoT security, intrusion detection, open-set recognition, unknown attack detection, prototype rejection, selective classification, sensor network traffic

## Abstract

Open-set IoT intrusion detection must distinguish unfamiliar attacks from uncertain known traffic. OpenTriIDS implements a three-action policy: a random forest accepts high-confidence predictions, routes uncertain samples to a compact known-class prototype memory, and rejects routed samples beyond a standardized Euclidean distance boundary. At the representative S1 operating points on Edge-IIoTset and CIC-IoT-2023, the operational macro-F1 reaches 68.44% and 76.81%, with routing rates of 4.53% and 4.13%, respectively. The representative S1 analysis shows that the confidence gate defines the selected subset, while the three-prototype support rule improves macro-F1 and changes the balance between known-class retention and unknown rejection; the magnitude and direction depend on the dataset and operating point. The prototype-distance module records the matched label and distance for each routed sample. A complementary controlled protocol evaluates three seeds across multiple held-out-family settings, while a seven-method S1 comparison evaluates MSP, OpenMax, ORI open recognition, EFC, post hoc Energy OOD, single-layer Mahalanobis scoring, and OpenTriIDS at their documented operating points. OpenTriIDS achieves the highest macro-F1 among the evaluated methods in both datasets under this comparison. Together, the analyses characterize a selective open-set policy whose matched-gate macro-F1 advantage is accompanied by dataset- and holdout-dependent unknown-recall trade-offs, while distinguishing predictive performance from routing and computational cost.

## 1. Introduction

IoT intrusion detection is commonly evaluated with the same attack taxonomy in training and testing, using benchmarks such as Edge-IIoTset and CIC-IoT-2023 [1,2,3,4]. Operational traffic can contain attack families absent from training. Because closed-set classifiers must assign these samples to existing labels, explicit unknown handling is needed alongside known-class recognition [5,6,7,8,9,10].

Low confidence can arise from unfamiliar attacks or overlap among known classes. Rejecting every uncertain prediction treats these situations identically. Recent open-world IDS methods use learned representations, calibrated scores, and inspection to recognize unknown traffic [11,12,13,14]. We ask whether known-class support can refine decisions within a fixed uncertain subset, and under which held-out-family conditions this refinement helps. OpenTriIDS separates confidence selection from final unknown assignment. The maximum posterior from the random forest selects uncertain flows; training-derived class-conditional prototypes then assess whether these flows are supported in standardized feature space. A routed sample is assigned the nearest prototype label when its distance is within a fixed boundary; otherwise, it is assigned to unknown. Unlike confidence-only rejection, this rule can retain uncertain known traffic without enlarging the selected subset. It extends selective classification by applying a support decision after selection [15,16], while the matched prototype and distance provide a traceable record. Section 2 distinguishes this conditional support decision from learned-density recognition and statistical or neural score calibration.

At the representative S1 operating points, OpenTriIDS reaches macro-F1 values of 68.44% on Edge-IIoTset and 76.81% on CIC-IoT-2023, with routing rates of 4.53% and 4.13%, respectively. On Edge, the prototype-distance module improves macro-F1 over confidence-only rejection while preserving the unknown recall. Across the controlled CIC settings, the module variants yield distinct macro-F1 values and a dataset-dependent balance between known-class retention and unknown recall. These findings emphasize explicit rejection and decision traceability, together with dataset- and holdout-dependent trade-offs in unknown recall. A complementary controlled analysis uses fixed paired rules for module ablations across multiple held-out-family settings, and a seven-method S1 comparison evaluates the documented operating points on shared partitions. OpenTriIDS achieves the highest macro-F1 among the evaluated methods in that comparison. This result is reported at the specified operating points; it is not presented as a matched-budget, runtime, or universal unknown-recall ranking. The representative and controlled analyses address distinct questions and are interpreted according to their respective protocols.

The contributions are:

- A known-class support decision after confidence gating, distinguishing uncertain known traffic from unsupported unknown traffic.
- A training-only, multi-prototype implementation with conditional distance scoring and a traceable nearest-prototype record.
- A representative working-point analysis with controlled evaluations across three seeds and multiple held-out-family settings and seven-method S1 comparisons that separate rejection benefits, prototype refinement, and operating conditions.

Section 2 reviews the related methods; Section 3 defines OpenTriIDS; Section 4 presents the protocol, results, and scope; and Section 5 concludes.

## 2. Related Work

### 2.1. IoT Intrusion Detection and Closed-Set Evaluation

Existing IoT intrusion-detection studies mainly investigate supervised machine-learning and deep-learning models using benchmark traffic datasets [9,17]. Their evaluation generally focuses on distinguishing benign traffic from attack classes represented in the training taxonomy. Public datasets such as Edge-IIoTset and CIC-IoT-2023 have enabled reproducible comparisons across IoT and industrial IoT (IIoT) traffic conditions [1,2].

However, the use of the same benchmark does not guarantee the same evaluation problem. Feature construction, data screening, class definitions, and partition rules can substantially change the assessed population [6]. Cross-evaluation studies further show that an IDS that performs well within one dataset or network context may not retain the same behavior under a different traffic distribution [8,10]. These findings motivate separating closed-set classification from open-set evaluation rather than treating them as interchangeable evidence of deployment readiness.

Closed-set evaluation assumes that test samples belong to classes represented during training. It therefore measures discrimination among known classes but does not directly test whether an attack family absent from training will be rejected as unknown [5,9]. Open-set and zero-day studies address this limitation by withholding attack classes, modeling uncertainty, estimating distance from known data, or introducing additional recognition and updating stages [7,11,18,19]. These protocols answer different questions: held-out-family evaluation measures unknown rejection under a fixed population, whereas cross-domain and online-learning settings additionally examine transfer and adaptation.

The present study follows the held-out-family line of evaluation. It uses public IoT/IIoT benchmarks while reserving selected attack families for testing only, allowing known-class recognition and unknown-attack rejection to be evaluated together. This protocol does not claim to establish cross-domain transfer or online continual learning. Instead, it provides the setting for examining whether confidence-selected traffic contains sufficient support for a known class before assigning it to an unknown category.

### 2.2. Open-Set and Zero-Day Intrusion Detection

Open-set recognition extends known-class classification with rejection beyond the training label vocabulary [20,21]. Related IDS research addresses unknown-attack discovery [7], zero-day and zero-shot detection [18,19], and open-set rejection using dissimilarity or an unsupervised structure [22,23,24]. These directions share an interest in unfamiliar traffic but differ in their supervision assumptions and output requirements. Open-world research additionally considers how rejected samples can be inspected or incorporated into subsequent learning [11,14,25]. Applications span CAN-bus recognition, IoT-specific networks, and cross-granularity intrusion detection [12,26,27]. Thi and Gurusamy’s MDCG-IDS represents traffic across statistical, temporal, and spatial dimensions at multiple granularities and uses optimal-transport distances in a semi-supervised, benign-only setting. This broadens the evidence available for open-set detection but differs from OpenTriIDS’s supervised known-class triage and conditional prototype scoring. [27].

A representation-and-neighborhood approach is exemplified by Du et al.’s ORI, which combines supervised contrastive embeddings, neighborhood-density recognition, and nearest-neighbor known classification [14]. Its inspection module subsequently relearns representations from known and recognized-unknown samples, obtains analyst labels for selected representatives, and applies constrained clustering. This extends recognition to the organization and labeling of rejected traffic but introduces an inspection stage that requires additional data and human input. OpenTriIDS addresses the earlier decision of whether uncertain traffic retains sufficient known-class support, using a frozen training memory without analyst-assisted updates.

Meta-recognition instead models evidence associated with known classes. Zhang et al. combine neural activation vectors, latent features, and reconstruction errors with class-specific extreme-value models, tail separation sampling, and temperature scaling [13]. Their method integrates multiple neural signals to support fine-grained known classification and explicit unknown output, and processes each test sample through the resulting distance and EVT-based scoring pipeline. OpenTriIDS uses training-standardized numerical features and conditional Euclidean prototype scoring. The distinction concerns the representation, rejection evidence, and samples receiving additional assessment, rather than the existence of a second decision stage.

Souza et al.’s Energy-based Flow Classifier (EFC) provides a statistical alternative by modeling dependencies among discretized features through class-conditional energies [28]. It rejects samples exceeding the minimum-energy class’s training-derived boundary. Its acceptance regions depend on discretization and estimated class distributions, rather than a learned embedding or Euclidean prototype geometry. EFC scores every sample and is distinct from neural logit-based energy scoring.

These approaches differ in representation, rejection rules, and post-rejection processing. Published results obtained under different protocols therefore do not directly establish a performance ranking. Section 4.4 compares independent ORI open recognition and EFC implementations with OpenTriIDS and four classical OOD/reference scores on common S1 partitions and three operational labels: benign, known attack, and unknown attack. Each method retains its documented scoring and calibration rule, so the comparison concerns predictive macro-F1 at the stated operating points rather than a matched rejection budget or runtime frontier. Zhang et al.’s method provides a technical point of comparison, whereas ORI’s analyst-assisted inspection lies outside the evaluated recognition task.

### 2.3. Selective Prediction and Confidence Quality

Selective classification returns a label or abstains through a classifier/selection pair. Geifman and El-Yaniv formulate confidence-threshold selection with a risk–coverage objective and probabilistic guarantees under their sampling assumptions [15]. SelectiveNet further integrates the reject option into network training [16]. OpenTriIDS uses confidence to select samples for an additional support decision, assigning them to a supported known class or unknown rather than simply abstaining. The selective classification guarantees are not transferred to held-out attacks.

Effective selection requires a score that ranks prediction risk, whereas calibration concerns the agreement between confidence and empirical correctness [15,29,30]. Nominal confidence may be unreliable under distribution shift, and calibration on known data does not by itself establish separation from unknown classes [31]. OOD research accordingly distinguishes score construction, calibration, and rejection under different evaluation assumptions [32,33,34].

Maximum softmax probability (MSP) uses the largest neural softmax output, while ODIN adds temperature scaling and input perturbation [35,36]. Energy OOD scores classifier logits through log-sum-exp, Mahalanobis scoring measures covariance-normalized feature distances, and OpenMax allocates explicit unknown probability [20,37,38]. These methods use different evidence for rejection; their scores and numerical thresholds are therefore not directly interchangeable. Our Energy and single-layer Mahalanobis adaptations share an MLP and known-validation calibration, retain their accepted-class predictions, and score every sample. They exclude energy fine-tuning/outlier exposure and Mahalanobis input perturbation/multi-layer fusion; Section 4.4 specifies the implementations.

The RF confidence-only control instead rejects samples whose maximum unmodified posterior is below a fixed threshold. Given identical posteriors, class mappings, thresholds, and comparison rules, maximum-probability rejection is the same control, not an independent baseline. MSP here denotes neural softmax scoring in the literature. Equal numerical thresholds across different detectors or scores need not produce equal rejection rates or comparable operating points.

### 2.4. Traceable Decisions in Cyber-Defense Settings

Trustworthy IDS research examines the reliability of predictions through uncertainty quantification [39,40]. Sander et al. provide a related example by combining neural packet-payload representations with XGBoost-based symbolic reasoning and comparing confidence scoring, Shannon entropy, and post hoc uncertainty metamodeling for open-set recognition [41]. These directions motivate considering both the predicted outcome and the information available for reviewing it. Uncertainty estimates describe aspects of predictive reliability, whereas decision traceability concerns the ability to inspect the information and rule used for a particular output. Neither alone establishes that an analyst can interpret an alert correctly or resolve it more efficiently.

OpenTriIDS provides a traceable support record for routed samples: the nearest prototype label and standardized Euclidean distance identify the known-class reference and its relation to the acceptance boundary. Optional standardized feature-deviation diagnostics indicate which measured attributes differ most from that prototype. These records describe proximity in the fitted feature space; they are not causal explanations of malicious behavior or evidence of improved analyst effectiveness.

Together, the literature motivates examining how uncertain known traffic can be retained while unsupported traffic is rejected. OpenTriIDS studies this question by separating confidence selection from conditional support assessment. Matched-gate ablations evaluate the resulting balance between known-class preservation and unknown-attack detection, while the prototype record makes the support decision inspectable. The usefulness of these records in analyst workflows requires a separate evaluation.

## 3. Method

### 3.1. Problem Formulation

Let $x\in R^{d}$ be a traffic vector standardized with feature-wise means and scales fitted on all retained-known training samples. The detector and prototypes share this frozen transformation. The internal label set $Y_{\mathrm{known}}$ contains benign traffic and retained attack families; evaluation uses Equation (1)

(1) $$Y_{\mathrm{op}}=\left\{\mathrm{benign},\;\mathrm{known}\;\mathrm{attack},\;\mathrm{unknown}\;\mathrm{attack}\right\}.$$

The base detector returns a predicted known-class label and a detector-side confidence score in Equation (2):

(2) $$\left(y_{\mathrm{base}},c_{\mathrm{base}}\right)=f\left(x\right),$$

where $y_{\mathrm{base}}\in Y_{\mathrm{known}}$ and $c_{\mathrm{base}}\in \left[0,1\right]$. The controller either accepts this label or routes the sample for prototype assessment.

The confidence threshold $\tau_{c}$ controls routing; the distance threshold $\tau_{u}$ controls acceptance around the nearest prototype $p^{★}$ in the known-class memory P, with the nearest-prototype rule defined in Section 3.4. The observed routing fraction *r* is a selection rate, not a measured computational budget.

### 3.2. Method Overview

**Figure 1:**
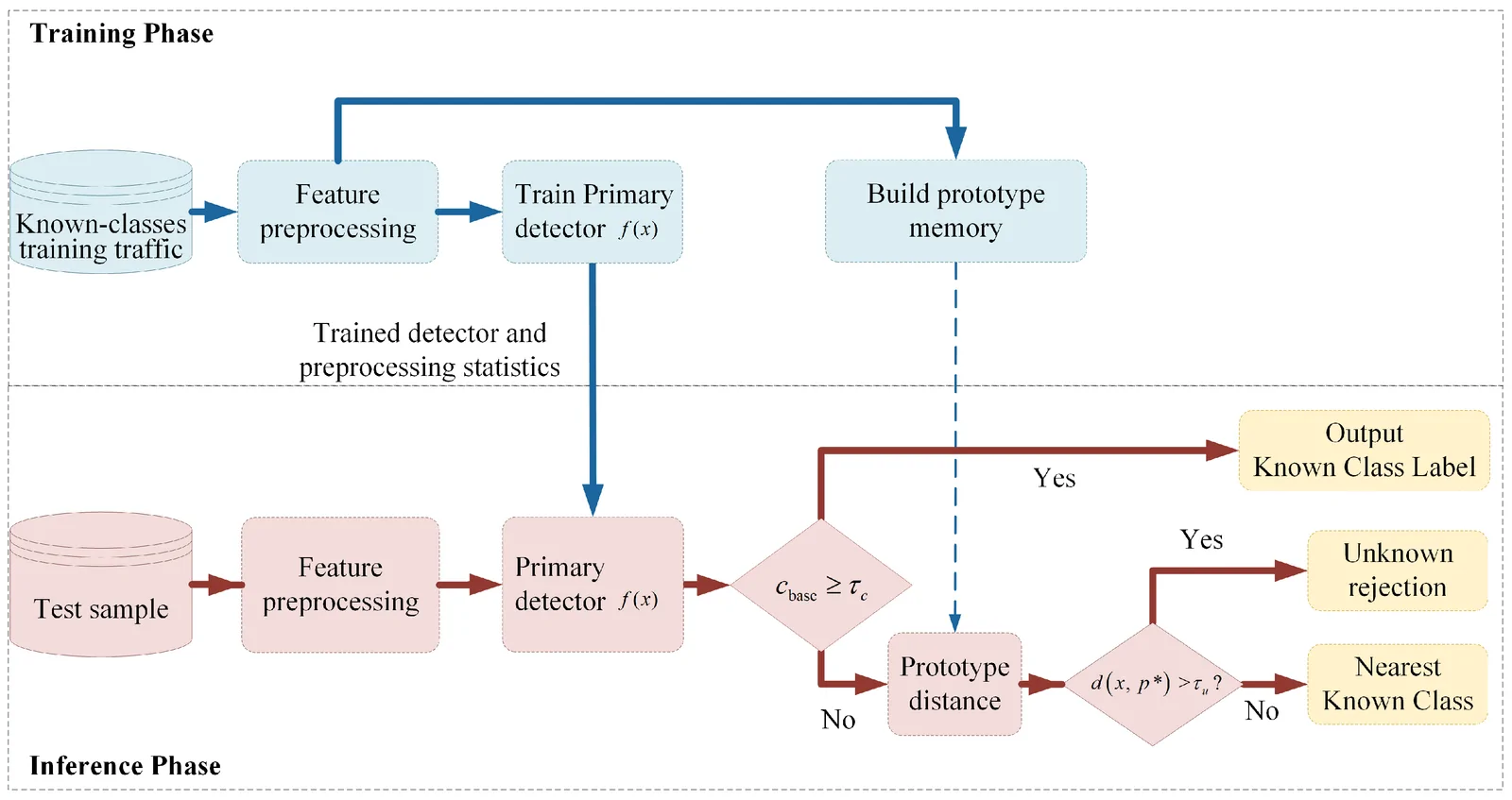
Selective open-set triage architecture.

Figure 1 summarizes the training and inference flow of OpenTriIDS. During training, known-class traffic is transformed with feature-wise statistics fitted from the retained-known training samples. The same frozen transformation is supplied to the primary detector and to the prototype memory, so their scores are expressed in one consistent feature space. During inference, each test sample first passes through the frozen preprocessing step and the primary detector. The detector output is then evaluated by a confidence gate: high-confidence samples are accepted directly, whereas low-confidence samples are selectively sent to the prototype-distance module. The nearest prototype provides a candidate known class and a distance score; the distance boundary either retains that candidate or assigns the sample to the explicit unknown class. Thus, the confidence gate determines when additional prototype assessment is activated, while the distance boundary determines whether the routed sample has sufficient support for a known-class assignment. The figure is a methodological overview; it does not itself define evaluation metrics or operating points.

Module A: primary detector

Each dataset–holdout–seed run fits one random forest (RF) on the retained-known training data. For every input, the RF produces both a predicted known-class label and its posterior confidence. These detector outputs are shared by the confidence-only control and all prototype-enabled variants, which isolates the effect of the subsequent support assessment from changes in the primary classifier.

Module B: uncertainty gate

The largest unmodified RF predict_proba entry determines routing. If the confidence is at least $\tau_{c}$, the detector label is accepted without further processing. If it is below $\tau_{c}$, the sample is routed to prototype scoring in the prototype-enabled variants and is assigned as unknown directly in the confidence-only control. The gate therefore creates a selective second-stage workload rather than replacing the primary detector, and the controlled evaluation uses the uncalibrated RF probabilities as the detector-side confidence.

Module C: prototype-distance module

The prototype memory is constructed only from training samples and stores up to three k-means centers for each known class. For a routed sample, distances to all stored centers are evaluated in the shared standardized space. The closest center determines both the candidate known label and the distance used by the final decision, allowing the module to represent multiple modes within a class while keeping inference restricted to the routed subset.

Module D: explicit unknown decision

The explicit unknown decision compares the nearest-prototype distance with $\tau_{u}$. A routed sample is assigned the nearest prototype label when the distance is within the boundary, including equality; otherwise, it is assigned to unknown. The resulting policy therefore has three operational actions—direct detector acceptance, prototype-supported known assignment, and unknown rejection—which are subsequently mapped to the benign, known-attack, and unknown-attack output labels used for evaluation.

### 3.3. Conditional Known-Class Support Assessment

The three actions are detector acceptance, prototype-supported known assignment, and unknown rejection. For fixed model parameters, the policy is given in Equation (3):

(3) $$\pi \left(x\right)=\left\{\begin{array}{cc} a_{\mathrm{acc}}, & c_{\mathrm{base}}\geq \tau_{c},\\ a_{\mathrm{kn}}, & c_{\mathrm{base}}<\tau_{c},\;d\left(x,p^{★}\right)\leq \tau_{u},\\ a_{\mathrm{unk}}, & c_{\mathrm{base}}<\tau_{c},\;d\left(x,p^{★}\right)>\tau_{u}. \end{array}\right.$$

All samples receive detector predictions; only the confidence-selected subset receives prototype scoring. Prototype-supported known assignment may produce either benign or known attack after label mapping; the three actions are distinct from the three operational output labels.

The confidence-only control is defined using the same detector output and gate in Equation (4):

(4) $${\hat{y}}_{\mathrm{conf}}\left(x\right)=\left\{\begin{array}{cc} y_{\mathrm{base}}, & c_{\mathrm{base}}\geq \tau_{c},\\ \mathrm{unknown}, & c_{\mathrm{base}}<\tau_{c}. \end{array}\right.$$

Maximum-probability rejection with identical RF posteriors, class mapping, and threshold rules is therefore the same control. In this manuscript, the RF maximum-posterior rule is called confidence-only rejection; it is distinct from neural maximum softmax probability (MSP), which is a separate score based on neural softmax outputs. Prototype variants change only post-gate assignments.

Prototype support retains some samples that confidence-only rejection would label unknown. This can avoid known false rejection or incorrectly retain unfamiliar attacks, motivating joint evaluation of known rejection, unknown recall, precision, and macro-F1.

### 3.4. Prototype-Distance Scoring and Unknown Rejection

The routing gate in Equation (5) is

(5) $$g\left(x\right)=\left\{\begin{array}{cc} 1, & c_{\mathrm{base}}<\tau_{c},\\ 0, & \mathrm{otherwise}. \end{array}\right.$$

When the gate is zero, the detector label is returned; when it is one, the prototype-distance module is invoked. The selected fraction varies by setting.

For each known class, k-means fits up to three labeled centers using standardized training samples. A routed sample is compared with every stored center according to Equation (6):

(6) $$p^{★}=\arg\min_{p\in P}d\left(x,p\right),$$

Here, d(·,·) is the Euclidean distance in the shared standardized space. Ties are resolved by stored order: sorted class labels, then the fitted k-means center order within each class.

The distance boundary then determines whether to reject the sample as unknown as specified in Equation (7):

(7) $$y_{\mathrm{proto}}\left(x\right)=\left\{\begin{array}{cc} \mathrm{unknown} & \mathrm{if}\;d\left(x,p^{★}\right)>\tau_{u},\\ \mathrm{label}(p^{★}) & \mathrm{otherwise}. \end{array}\right.$$

Equality is accepted. Combining this rule with the gate gives the internal class-or-unknown prediction in Equation (8):

(8) $$\hat{y}\left(x\right)=\left\{\begin{array}{cc} y_{\mathrm{base}}, & g(x)=0,\\ y_{\mathrm{proto}}\left(x\right), & g(x)=1. \end{array}\right.$$

Distances are interpreted within each dataset’s standardized representation; their numerical scale is not universal across datasets or protocols.

### 3.5. Data Preprocessing and Training

This subsection separates data construction from model fitting. Algorithms 1 and 2 specify the controlled preprocessing and training procedure. Conflict screening uses the complete input data and their labels before partitioning, thereby defining the evaluated population. The subsequent fitting of the scaler and models uses retained-known training samples only. The controlled methods share the cleaned rows and partitions, whereas their method-specific transformations are defined by the corresponding implementations. The sample counts and implementation settings below apply only to the controlled protocol.
**Algorithm 1** Data preprocessing and partitioning for the controlled evaluation**Require:** Labeled traffic data; fixed ordered numerical features; held-out attack families; random seed
  1:Extract the selected features, excluding label and exported-index columns  2:Normalize class labels: trim whitespace, uppercase, and remove non-alphanumeric characters  3:Convert features to numeric values; remove rows with missing/empty labels, conversion failures, or non-finite values, without imputation  4:Group rows by identical selected-feature vectors; remove every row in groups with conflicting class labels  5:Retain the first source row in each remaining exact-duplicate group  6:Separate retained-known rows from held-out attack rows; reserve all held-out rows for testing  7:Shuffle and stratify known rows by class: reserve 20% for testing using the run seed  8:Stratify the remaining 80% into training and validation at 75:25 using the same seed  9:Require every retained-known class in training, validation, and known testing10:Combine known-test and held-out rows; sort each partition by source-row order11:Fit feature means and population standard deviations on known training rows only; use unit scale for zero variance12:Standardize training and validation features; retain the same frozen transform for test-time inference13:**return** standardized training and validation sets; unscaled test rows with labels; frozen scaler and partition indices


**Algorithm 2** Training-stage construction for the controlled fixed-threshold evaluation**Require:** Standardized known-training and validation sets and frozen scaler from Algorithm 1; seed; prototype count 3; confidence threshold 0.75; distance threshold 1.7
  1:Fit one random forest on transformed training features and known labels  2:**for** each retained known class in sorted label order **do**  3:      Store its mean transformed training feature vector for the centroid ablation  4:      Fit class-conditional k-means with up to three centers on its transformed training samples  5:      Store the centers and their class label for the three-prototype variant  6:**end for**  7:Assign the prescribed thresholds; compute known-validation diagnostics without selection or refitting  8:Freeze the scaler, detector, prototype memories, and thresholds before test evaluation  9:**return** the frozen scaler, detector, two labeled prototype memories, and thresholds


Algorithm 2 fits the RF and prototype memories on standardized known-training samples. The controlled protocol uses a confidence gate of 0.75 and a distance boundary of 1.7 as fixed settings across datasets, holdouts, and seeds. This fixes the rule for paired comparisons rather than selecting a best test score. Equality is accepted at both boundaries. In this protocol, known-validation data provide routing and false-rejection diagnostics only; no grid search, cross-validation, or threshold optimization are performed.

For the controlled evaluation, the random forest uses 200 trees, Gini impurity, no maximum depth, a minimum of two samples for splitting and one sample per leaf, square-root feature subsampling, bootstrap sampling, and no class weights. Its random state is the run seed, and one CPU worker is used. Per-class k-means uses k-means++ initialization, 10 restarts, at most 300 iterations, the Lloyd algorithm, and a random state equal to the run seed plus the zero-based class index in sorted label order. The number of centers is capped by the number of distinct training vectors in that class; a single-center memory is the class mean. The implementation uses scikit-learn 1.7.2.

The three-prototype memory represents multiple class regions while keeping memory bounded, and Euclidean distance measures proximity in the standardized space. These fixed design choices are evaluated against a single centroid at the same boundary; they are not presented as search-selected optima. Their effects depend on feature geometry; other counts and distances remain untested.

### 3.6. Inference Procedure and Label Mapping

Algorithm 3 returns either a known-family label or an unknown; the operational mapping is applied afterward.
**Algorithm 3** Selective open-set inference with prototype-distance rejection.**Require:** Numerical sample v, frozen scaler *S*, detector *f*, confidence threshold $\tau_{c}$, labeled prototype set P, distance threshold $\tau_{u}$
  1:x←S(v)  2:$\left(y_{\mathrm{base}},c_{\mathrm{base}}\right)\leftarrow f\left(x\right)$  3:**if** $c_{\mathrm{base}}\geq \tau_{c}$ **then**  4:      **return** $y_{\mathrm{base}}$  5:**end if**  6:Retrieve nearest prototype $p^{★}\in P$  7:Record the matched label and distance $d(x,p^{★})$  8:Optionally export the five largest absolute feature deviations; prediction is unchanged  9:**if** $d\left(x,p^{★}\right)>\tau_{u}$ **then**10:      **return** unknown11:**else**12:      **return** $\mathrm{label}(p^{★})$13:**end if**


Predictions map benign labels to benign, retained attack labels to known attack, and explicit unknown outputs to unknown attack. Ground truth is mapped separately, with held-out families assigned as unknown attack. A held-out sample predicted as a known family therefore remains an error. This preserves fine-grained internal labels while evaluating the three-class operational decision.

### 3.7. Additional Scoring and Interpretation

For *K* known classes, up to *m* prototypes per class, and *d* features, memory and per-routed-sample scoring require O(Kmd). At routing fraction *r*, the average additional distance computation is O(rKmd). Confidence-only rejection performs no distance scoring; centroid and three-prototype variants use different memory sizes at the same gate. End-to-end cost also includes preprocessing, detector inference, implementation, batching, and hardware. Matching the gate or rejection rates therefore does not establish equal runtime or cost optimality.

The matched label and distance provide decision traceability. The optional export records the five largest absolute standardized feature deviations; it does not affect predictions and is excluded from inference timing. These are proximity diagnostics, not causal explanations; analyst effectiveness and deployment savings require separate evaluation.

## 4. Experiments

### 4.1. Experimental Setup

The experiments provide two complementary evaluations. The representative S1 analysis reports the designated working point and headline values, whereas the controlled analysis evaluates matched-gate ablations over two datasets; three held-out-family settings; and seeds 42, 1234, and 2025. The controlled variants share the fitted random forest, preprocessing, posterior array, and test samples. Separate S1 comparisons assess complete pipelines under common partitions and metrics but method-specific training and rejection rules.

Edge-IIoTset supports IoT/IIoT cybersecurity research [1]. CIC-IoT-2023, released by the Canadian Institute for Cybersecurity at the University of New Brunswick, comprises benign traffic and 33 attack types from 105 IoT devices [2]. The representative S1 analysis follows its benchmark-specific feature spaces, with 40 numerical Edge variables and 39 numerical CIC variables. The controlled protocol uses 32 Edge numerical features, excluding labels, addresses, raw text, categorical/mixed-type fields, observed constants, and udp.stream, and 39 CIC numerical features, excluding the label. Each schema was fixed before evaluation and without test-score selection.

For the controlled protocol, applying Algorithm 1 retains 138,731 Edge samples across 15 classes and 494,128 CIC samples across 34 classes. Table 1 summarizes this screening. All retained rows are used without further subsampling. Cleaning changes class proportions, so the controlled findings concern the screened benchmark populations.

The held-out pairs are S1: MITM and Fingerprinting, S2: Backdoor and Port_Scanning, and S3: SQL_injection and Uploading for Edge-IIoTset. For CIC-IoT-2023, S1 withholds MITM-ARPSPOOFING and DNS_SPOOFING, S2 withholds RECON-OSSCAN and RECON-PORTSCAN, and S3 withholds DICTIONARYBRUTEFORCE and SQLINJECTION. Every retained row of these held-out classes enters the test set only, so these classes contribute no training or validation samples.

### 4.2. Representative S1 Results

**Figure 2:**
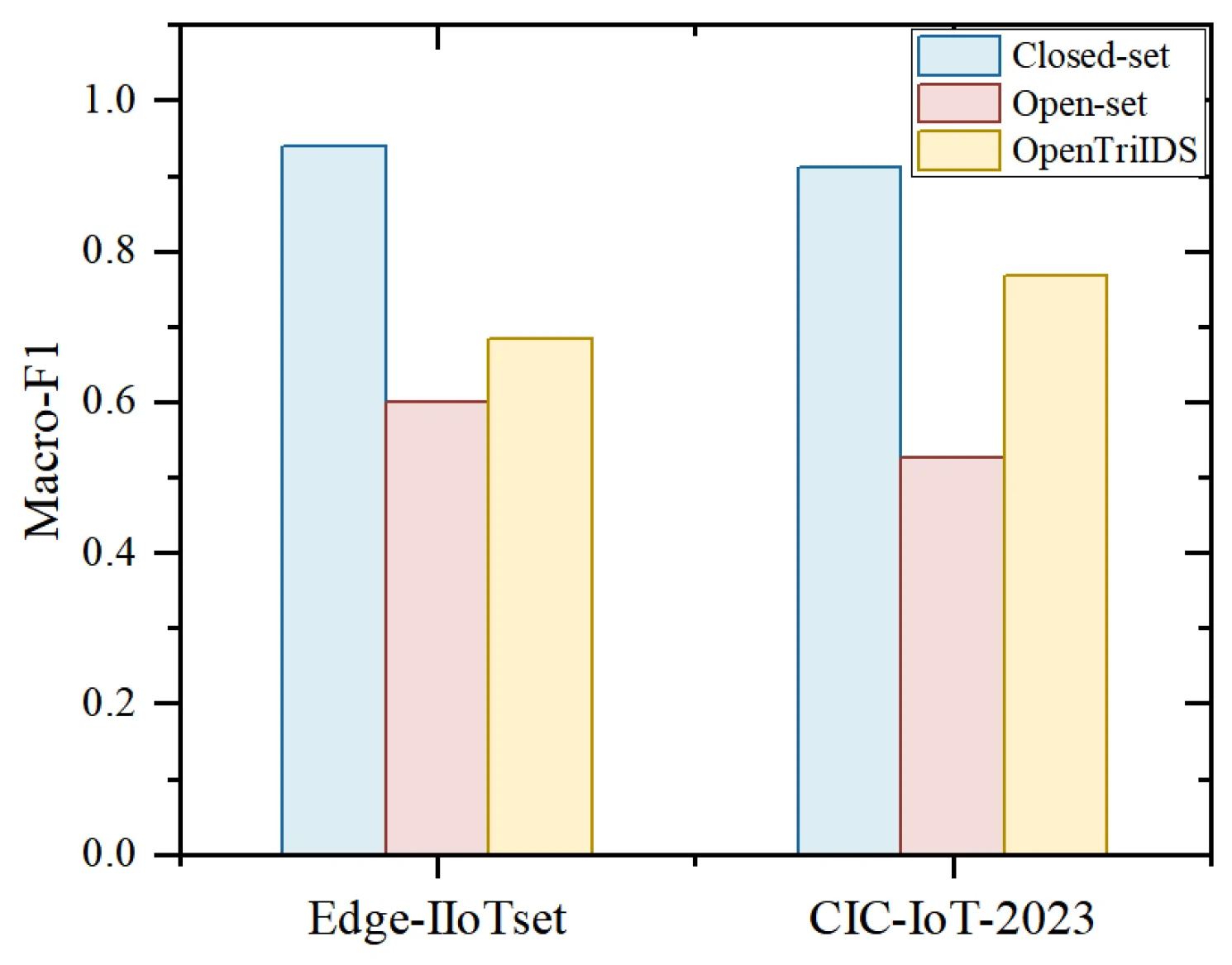
Representative S1 macro-F1.

**Figure 3:**
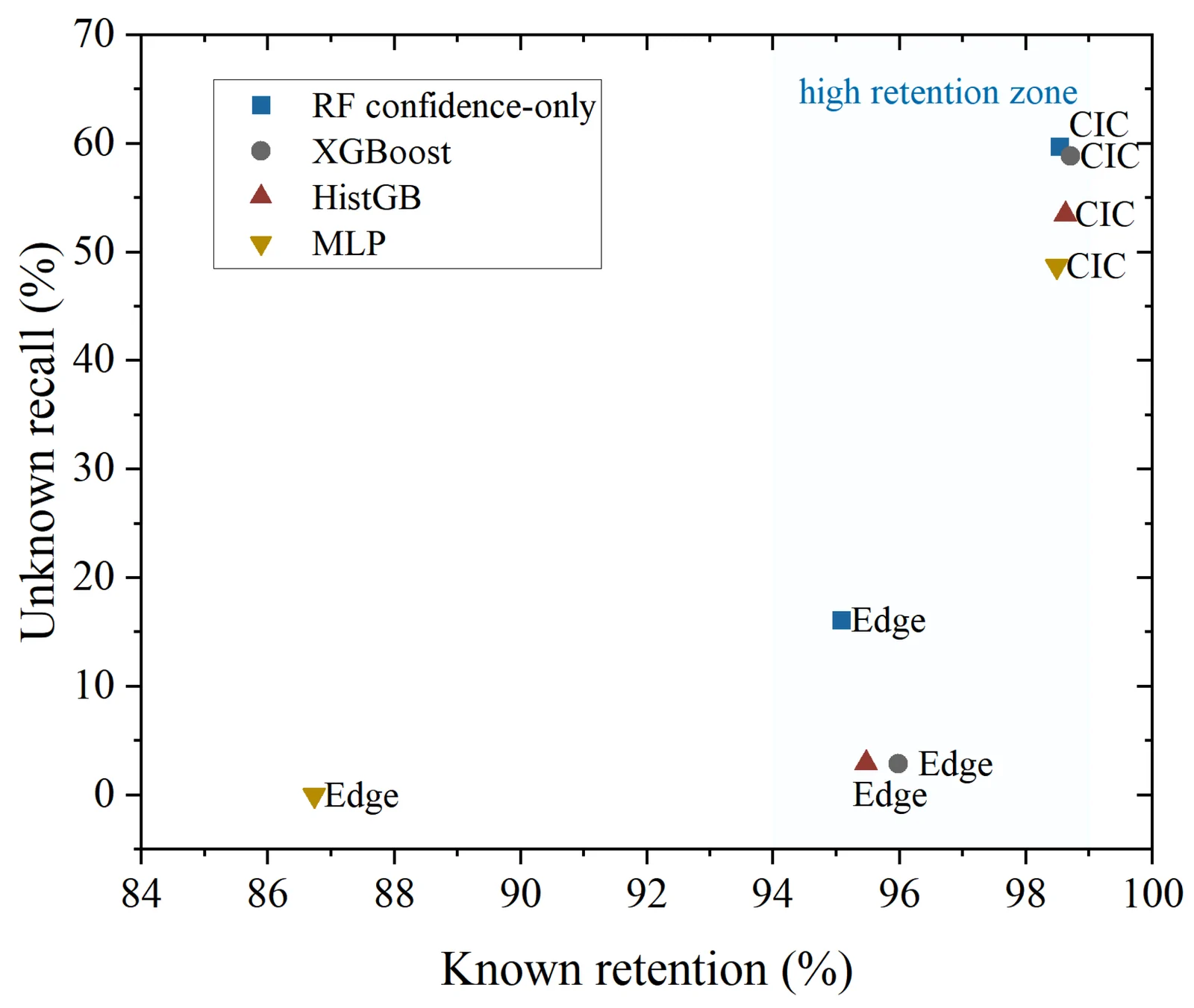
S1 known retention versus unknown recall.

The representative S1 results use the designated protocol with a confidence threshold of 0.75 and distance threshold of 1.7. For this representative protocol, the working-point thresholds are selected using the training-stage held-out validation partition for the corresponding S1 setting, then frozen before test evaluation; neither held-out test labels nor test scores enter their selection. Table 2 reports this single-run operating point alongside the confidence-only and centroid controls. Figure 2 summarizes the representative macro-F1 values, whereas Figure 3 focuses on known retention and unknown recall for the four detector controls with defined values for both measures: RF confidence-only, XGBoost with explicit rejection, HistGB with explicit rejection, and MLP with explicit rejection. In Figure 3, the RF point therefore denotes the confidence-only control listed in Table 2; the closed-set and open-set RF baselines are not plotted because unknown recall is not applicable to these outputs. The distance-boundary sweep and training-stage validation retuning reported later provide sensitivity controls without replacing this designated working point.

At this representative point, Edge S1 routes 4.53% of test samples; confidence-only rejection and OpenTriIDS both achieve an unknown recall of 16.12% for the held-out attacks. Prototype refinement raises unknown precision from 23.63% to 28.02% and known retention from 96.29% to 97.05%. CIC S1 reports 4.13% routing and 59.73% unknown recall for all three variants; OpenTriIDS has 98.76% known retention and 71.46% unknown precision. The controlled results below extend this view with robustness across seeds and attribution under a fixed nominal rule.

### 4.3. Controlled Evaluation and Module Ablation

Known samples are stratified by class into training, validation, and testing at approximately 6:2:2 using seeds 42, 1234, and 2025. The two-stage split in Algorithm 1 uses scikit-learn train_test_split; integer rounding determines the exact sizes. Partitions are row-based rather than session- or device-separated. Table 3 reports known and unknown partition totals, which are identical across seeds, although row membership changes.

Section 3.5 specifies training-only standardization and model fitting. For the controlled analysis, the nominal thresholds 0.75 and 1.7 are retained as fixed protocol settings across datasets, holdouts, and seeds; no test scores or labels are used to adjust them. Comparator rules appear in Section 4.4. The module runs use Python 3.12.14, NumPy 2.1.2, pandas 2.3.3, SciPy 1.16.2, and scikit-learn 1.7.2.

The implementation, feature definitions, split records, and processed experimental materials are available from the corresponding authors upon reasonable request.

Three variants isolate the module’s contribution:

- Confidence-only rejection assigns every sample with maximum RF posterior below 0.75 to unknown.
- Centroid rejection applies the same gate, then accepts the nearest known-class mean if its distance is at most 1.7; otherwise it assigns it as unknown.
- OpenTriIDS uses the same rule with up to three k-means prototypes per known class.

All variants derive labels and confidence from one cached, unmodified RF posterior array. Checks across 18 runs confirm identical test-row order, archived maximum confidences and gate masks, unchanged detector labels outside the gate, and confidence-only unknown assignments equal to the gate. Confidence of exactly 0.75 remains accepted, as specified in Equation (4).

The routing rate and final unknown-assignment rate divide selected samples and unknown outputs, respectively, by all test samples. They coincide for confidence-only rejection, which performs no distance scoring. For prototype variants, the routing rate measures distance-scored traffic, whereas the unknown-assignment rate can be lower. OpenTriIDS’s S1 routing and unknown-assignment rates are 13.02%/4.33% on Edge and 30.40%/10.22% on CIC. Neither rate alone measures computational cost.

Operational macro-F1 averages the benign, known-attack, and unknown-attack F1 scores, assigning zero as undefined F1. Unknown recall divides correctly rejected held-out attacks by all held-out attacks; unknown precision divides the same numerator by all unknown assignments, or is zero when none occur. Known false rejection counts retained-known samples assigned unknown over all retained-known test samples (benign and attacks). Known retention equals one minus this rate, not classification accuracy. The normal false-alarm rate counts benign samples predicted as a known attack or unknown over all benign samples, whereas known-attack false rejection counts retained attacks assigned as unknown over all retained attack test samples.

Each metric is computed per seed and then summarized by the arithmetic mean and sample standard deviation (variance denominator of two) without pooling predictions. Textual performance values use these means unless explicitly stated otherwise; sample counts are exact integers. Percentage-point gains use unrounded paired metrics, allowing 0.01-point differences from subtracting printed values. Repeated S1 entries reuse the same runs. Seed variation is descriptive; holdout comparisons also change known classes and unknown prevalence.

#### Matched-Gate Module Ablation

**Figure 4:**
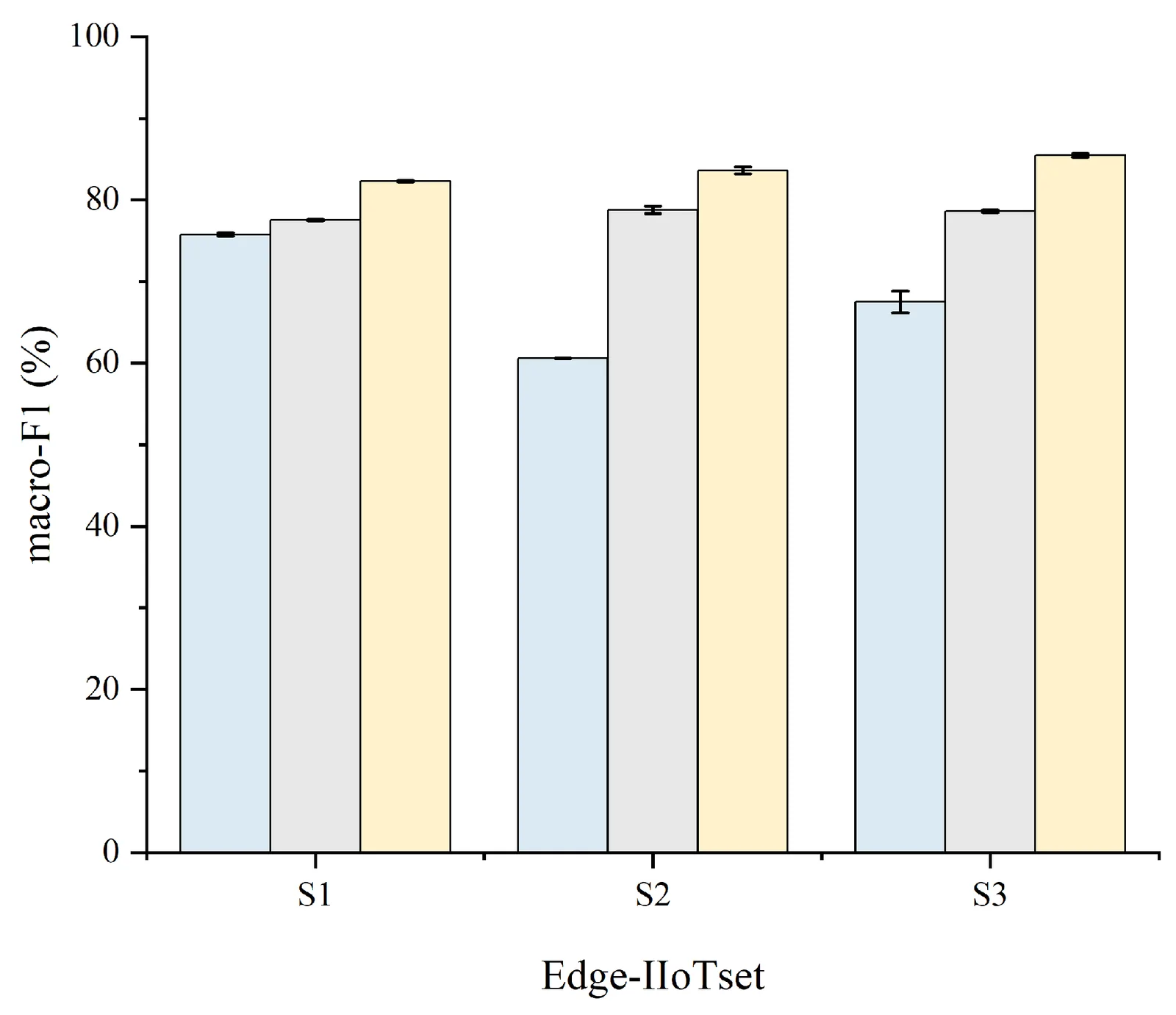
Matched-gate operational macro-F1 on Edge-IIoTset across S1–S3.

**Figure 5:**
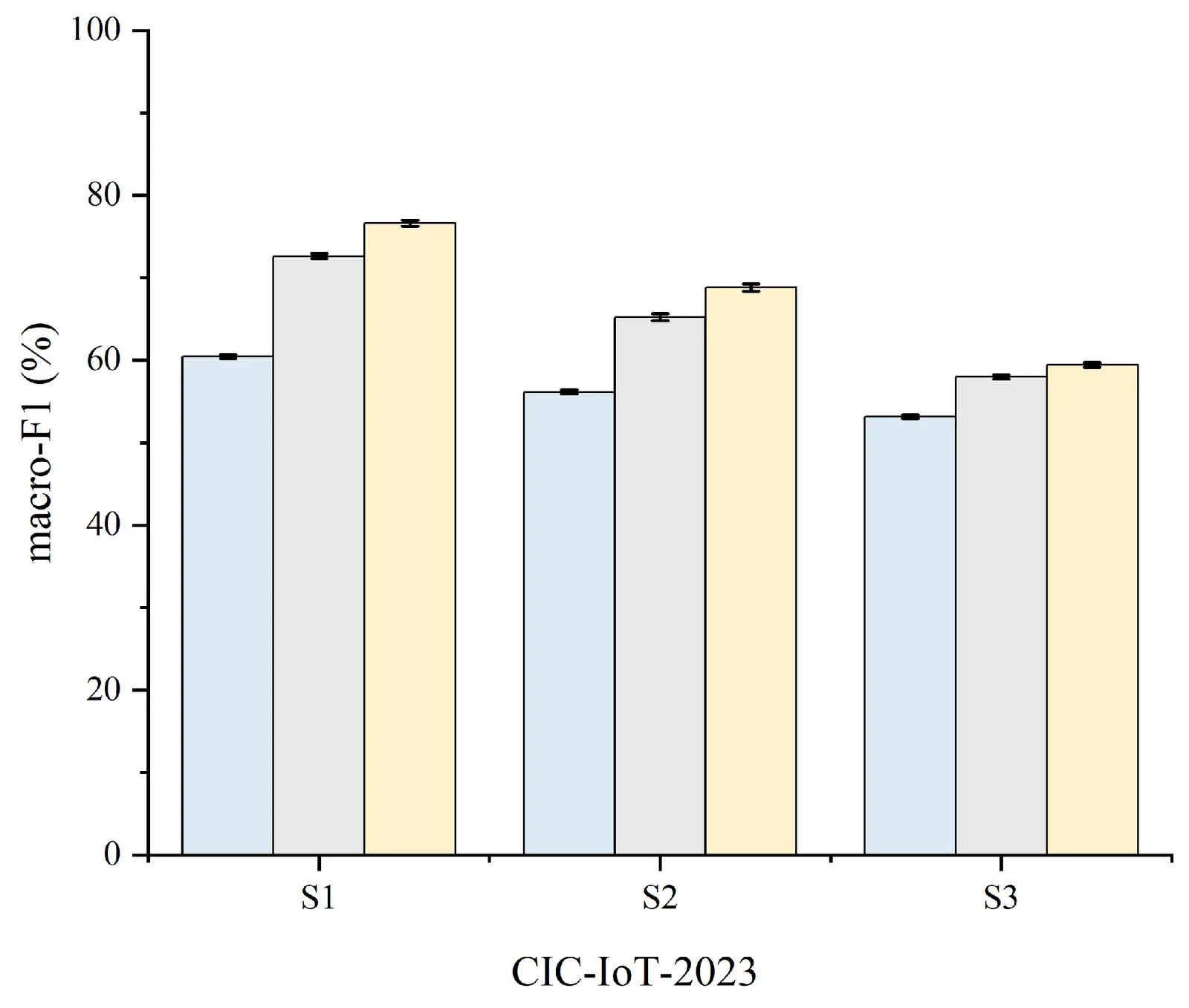
Matched-gate operational macro-F1 on CIC-IoT-2023 across S1–S3.

Figure 4 and Figure 5 report operational macro-F1 across S1–S3. Bars and error bars use the same per-seed results as Table 4.

The two macro-F1 figures show that OpenTriIDS has the highest mean macro-F1 in all six matched-gate settings. CIC S1–S3 show mean macro-F1 gains of 16.18, 12.66, and 6.27 percentage points over confidence-only rejection, and 3.98, 3.60, and 1.44 points over centroid rejection; the direction is consistent in every paired seed.

**Figure 6:**
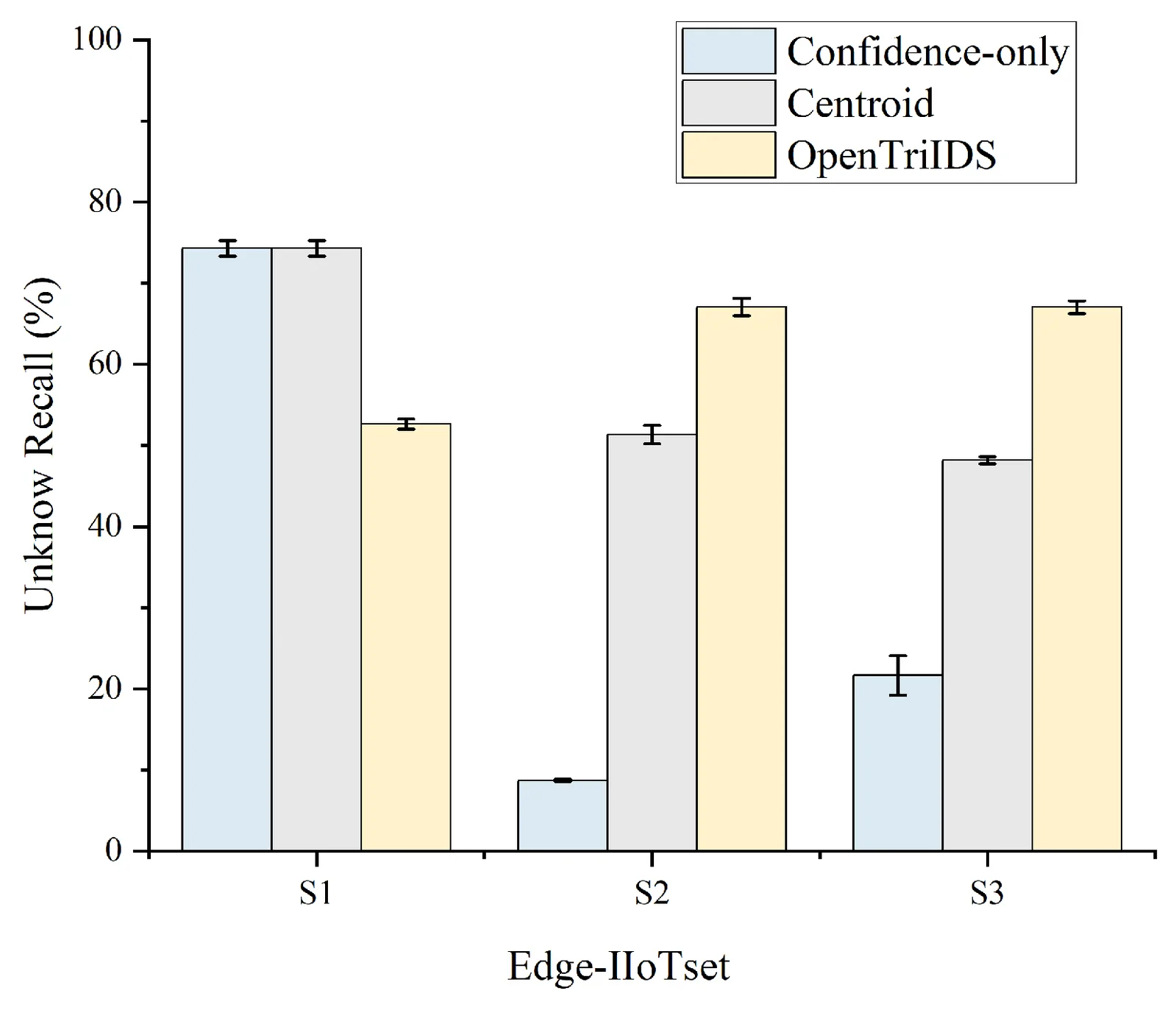
Matched-gate unknown recall on Edge-IIoTset across S1–S3.

**Figure 7:**
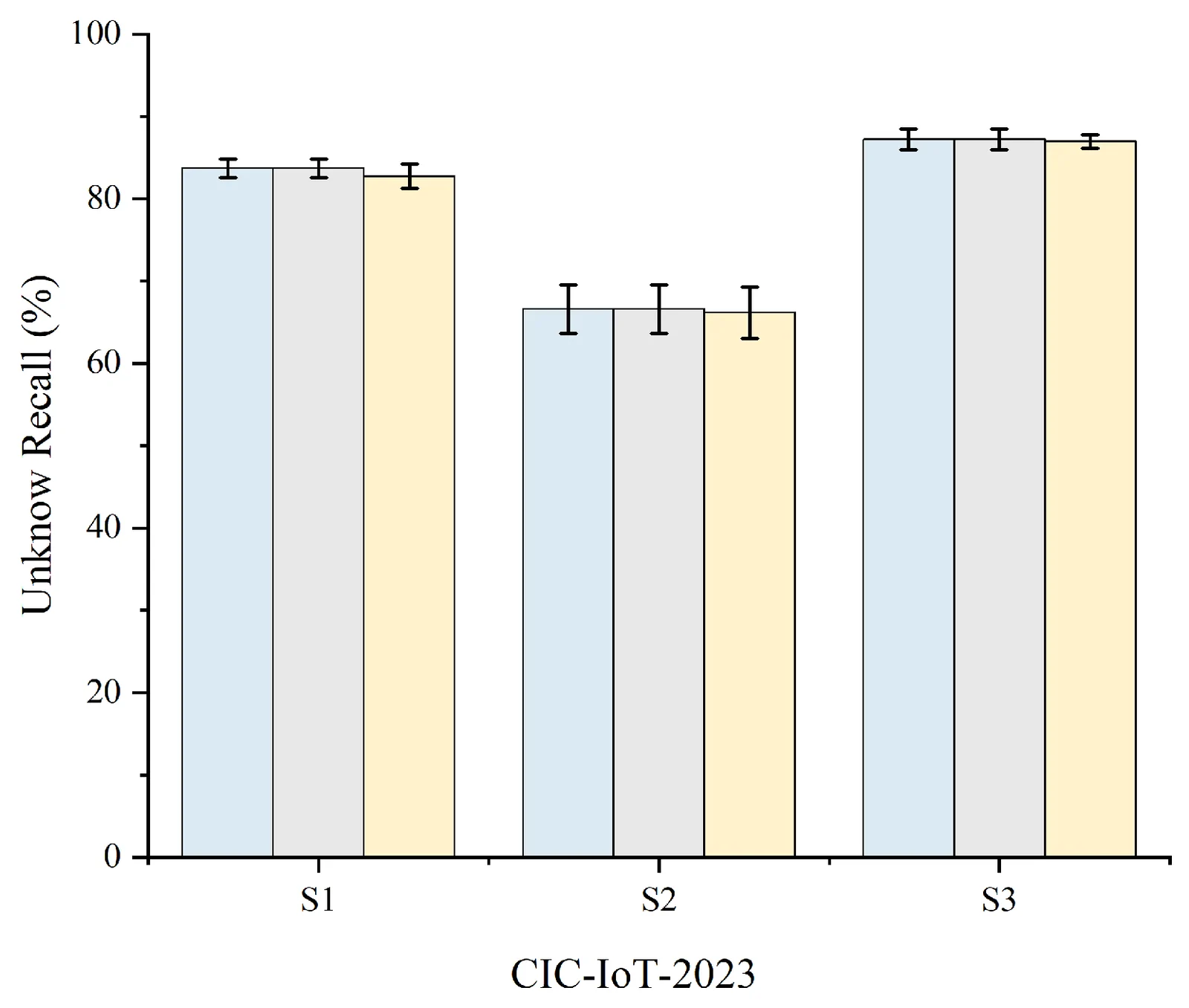
Matched-gate unknown recall on CIC-IoT-2023 across S1–S3.

The next two figures report unknown recall under the same matched gate, see Figure 6 and Figure 7.

On Edge, the macro-F1 advantage is accompanied by lower unknown recall than the two ablations, making the endpoint trade-off explicit. On CIC, unknown recall remains similar across the three variants for S1–S3.

The values in Table 4 are arithmetic means and sample standard deviations from the three-seed controlled protocol. They are therefore distinct from the single-run representative S1 values in Table 2, which use the designated representative protocol and its validation-selected working point. No representative single-run value is substituted for a controlled mean.

OpenTriIDS has the highest controlled macro-F1 in all three Edge settings. Its means are 82.30%, 83.60%, and 85.45% for S1–S3 compared with 75.73%, 60.59%, and 67.50% for confidence-only rejection. The largest gains therefore occur in S2 and S3. The unknown-recall panels show lower OpenTriIDS recall than both ablations across the Edge settings, so the macro-F1 advantage reflects a trade-off rather than uniform improvement on every endpoint.

### 4.4. Trade-Offs and External Method Comparison

#### 4.4.1. Known-Class Recovery and Unknown-Rejection Trade-Offs

Table 5 quantifies the trade-off. On Edge S1, three prototypes lower known false rejection from 10.33% to 2.21% and raise unknown precision from 23.99% to 51.07% relative to confidence-only rejection, while recall falls from 74.29% to 52.64%. Reduced known rejection therefore accompanies additional missed unknown attacks.

CIC S1–S3 show substantial reductions in known false rejection with comparatively small mean unknown-recall reductions. Centroid rejection provides an intermediate result; three prototypes further preserve known traffic within the same gate.

For Edge S2 and S3, three-prototype unknown recall falls to 8.71%/21.63% versus 67.06%/67.04% for confidence-only rejection. Some unknown attacks may bypass assessment through high confidence or receive known labels inside the prototype boundary. Confidence-only recall therefore provides an upper bound for prototype-refinement recall under the same gate. Feature overlap and the fixed boundary are plausible contributors to within-gate errors, but their individual causal effects are not isolated.

Although the ablations differ in known-attack and unknown outputs, they have identical normal-traffic alarm rates within each seed. Table 6 separates this shared rate from known-attack false rejection, which decreases with prototype refinement.

CIC S3 contains only 281 held-out attacks among 99,051 test samples. OpenTriIDS reaches 86.95% unknown recall but 3.98% precision, illustrating why higher module-level scores must be interpreted alongside prevalence, false alarms, and alert quality.

**Figure 8:**
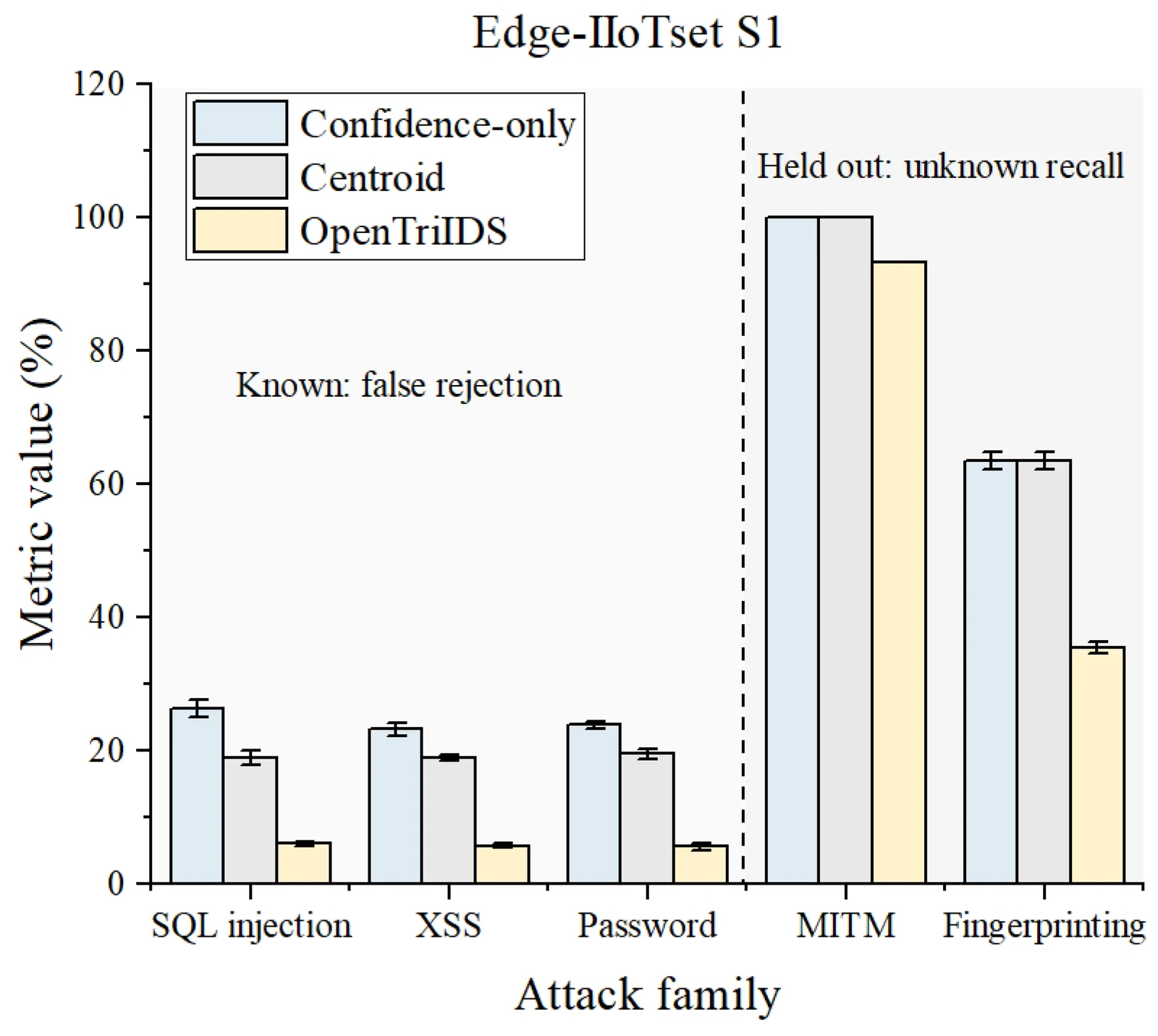
Class-level rejection diagnostics on Edge-IIoTset S1.

**Figure 9:**
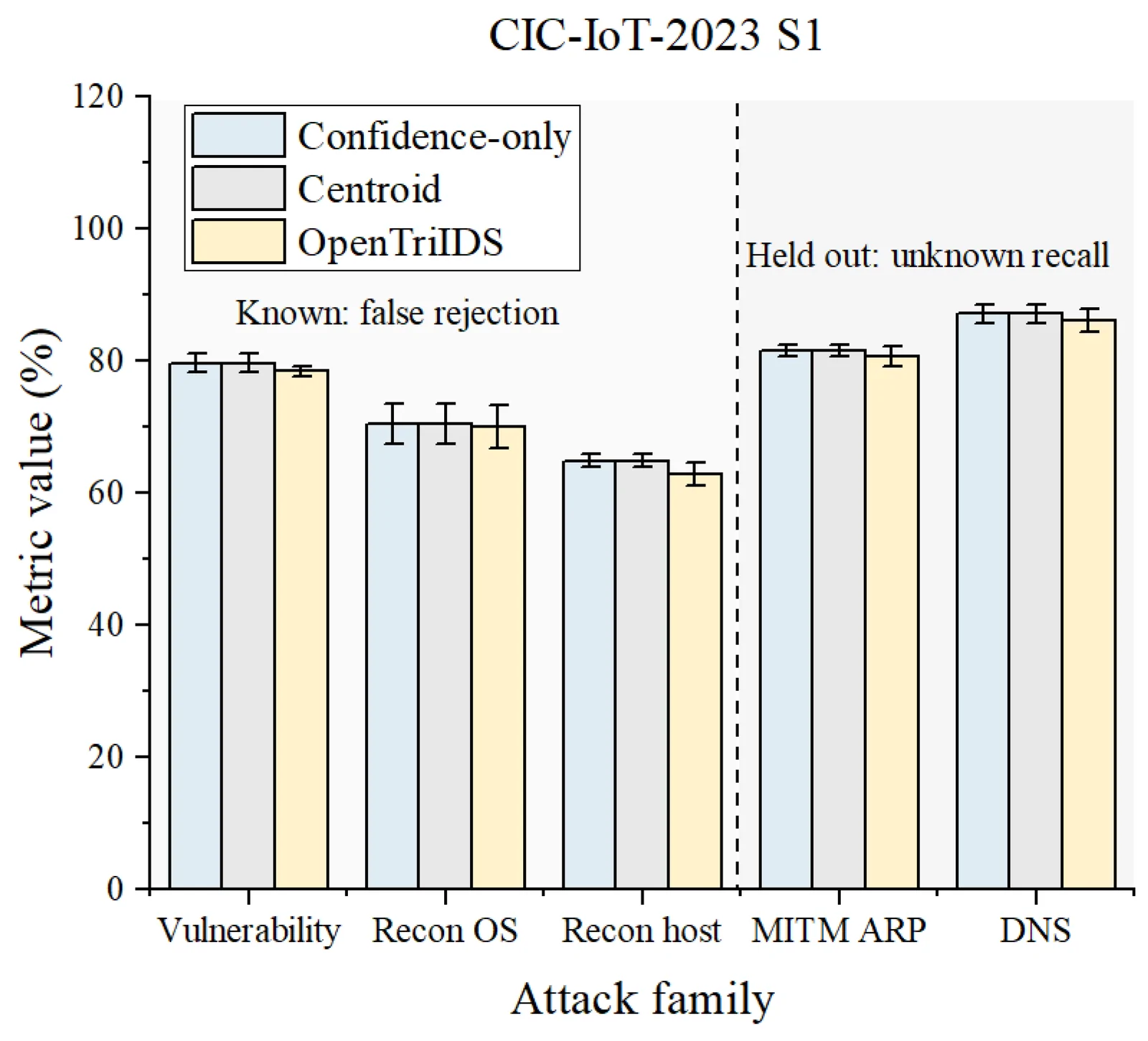
Class-level rejection diagnostics on CIC-IoT-2023 S1.

The next two figures examine three selected retained-known families and both held-out families per dataset on S1, see Figure 8 and Figure 9.

On Edge, prototype refinement reduces false rejection for the displayed known attacks, while unknown recall is 93.28% for MITM and 35.57% for Fingerprinting. On CIC, the displayed known families still incur 62.81–78.45% false rejection under OpenTriIDS. Thus, aggregate known-class recovery does not imply uniform family-level retention. These diagnostics use the frozen predictions underlying Table 5.

#### 4.4.2. External Method Comparison

The S1 comparison includes maximum softmax probability (MSP) and OpenMax as classical reference scores [20,35], together with ORI open recognition (ORI-OR) and multi-class open-set EFC [14,28], post hoc Energy OOD, and single-layer Mahalanobis scoring [37,38]. All seven methods are evaluated on the same S1 partitions, selected feature columns, label mappings, and operational metrics. Each method retains its specified preprocessing, scoring, and rejection rule; the corresponding details are given below. ORI-OR excludes analyst-assisted inspection and model updates.

MSP and OpenMax are evaluated under the same S1 protocol. Table 7 summarizes the macro-F1 means and sample standard deviations for all seven methods; routing, precision, and cost are not comparison criteria in this table.

ORI-OR uses training-only standardization and a ReLU embedding network with hidden widths 64/32 and 16 output dimensions. Supervised contrastive training uses unit-normalized embeddings, a temperature of 0.07, Adam (learning rate of 0.007, zero weight decay), a batch size of 256, and 100 epochs. The recognition radius averages each training sample’s mean distance to its 50 nearest training neighbors, excluding itself. Following the source Algorithm 1 in Du et al. [14], having at most five neighbors inside the radius implies unknown; otherwise, 25-neighbor majority voting assigns a known class. The implementation follows Algorithm 1 in Du et al. [14] when it differs from the accompanying prose. Neighbor ties follow training-row order; vote ties follow sorted classes. The numerical-input architecture, unit normalization, and exact float64 neighbor search are explicit adaptations. No analyst labels or rejected test samples enter training.

EFC uses training-only MaxAbs scaling and full-training linear-quantile discretization, with up to 30 bins per feature and no subsampling. Duplicate quantiles reduce bin counts; out-of-range values enter endpoint bins. Class energies follow Equations (5)–(8) in Souza et al. [28], with a pseudocount weight of 0.5, different-feature interactions, and all local-field terms. After selecting the minimum-energy class, rejection occurs strictly above its training boundary: an ascending energy element floor (0.95n) indexed from zero for n class samples. Equality is accepted as in Algorithm 1 of that reference; energy ties follow sorted classes. This equation-based implementation differs from older public routines in energy indexing and equality handling.

Energy and Mahalanobis share one MLP per dataset/seed: standardized inputs, ReLU hidden layers of widths 64/32, and a known-class head. Unweighted cross-entropy training uses Adam (learning rate of 0.001, weight decay of 0.0001), a batch size of 1024 including the incomplete batch, and 100 epochs. The final epoch is retained, without resampling, dropout, early stopping, or validation checkpoint selection. Seeds control initialization and shuffling. The last hidden layer is not L2-normalized. These are fixed numerical-input adaptations, not source-paper IoT-tuned settings.

Energy uses raw logits $z_{k}$ to compute $E\left(x\right)=-T\log\sum_{k}\exp\left(z_{k}\left(x\right)/T\right)$, with T=1; larger scores indicate unknownness. This post hoc rule uses neither outlier exposure nor energy fine-tuning. Mahalanobis fits class means and a shared within-class covariance on all training embeddings, dividing by the training sample count. Its diagonal ridge is $\max(10^{-12},10^{-6}\mathrm{tr}\left(\Sigma \right)/32)$; the score is the minimum squared class distance. It uses no input perturbation or multi-layer fusion. Both methods retain the shared head’s argmax label for accepted samples, with sorted-class tie-breaking.

Both OOD cutoffs use a prespecified 95% known-validation retention target. The cutoff is the ascending-score element $\lceil 0.95n_{\mathrm{val}}\rceil -1$ using zero-based indexing. Only strictly larger scores are rejected, ensuring at least 95% empirical validation retention rather than a fixed test rate. Validation updates neither network nor Gaussian statistics; test scores and labels select no parameters.

Thus, OpenTriIDS, ORI-OR, Energy, and Mahalanobis share training-standardized inputs, while EFC transforms the same columns independently. OpenTriIDS has fixed thresholds; ORI-OR/EFC derive training boundaries and use validation diagnostically; only Energy/Mahalanobis share the 95% validation target. Hyperparameters are fixed across datasets/seeds; fitted parameters are run-specific. Partition identity, feature order, and reference preprocessing records were verified for every comparator run.

These results characterize the predictive operating profile of each method under its documented decision rule. The methods use method-specific operating points rather than a matched rejection rate or computational budget; the external comparators score every sample without RF routing, so routing is not applicable rather than zero. The comparison therefore supports a macro-F1 comparison at the stated operating points, not an accuracy–cost or runtime ranking.

**Figure 10:**
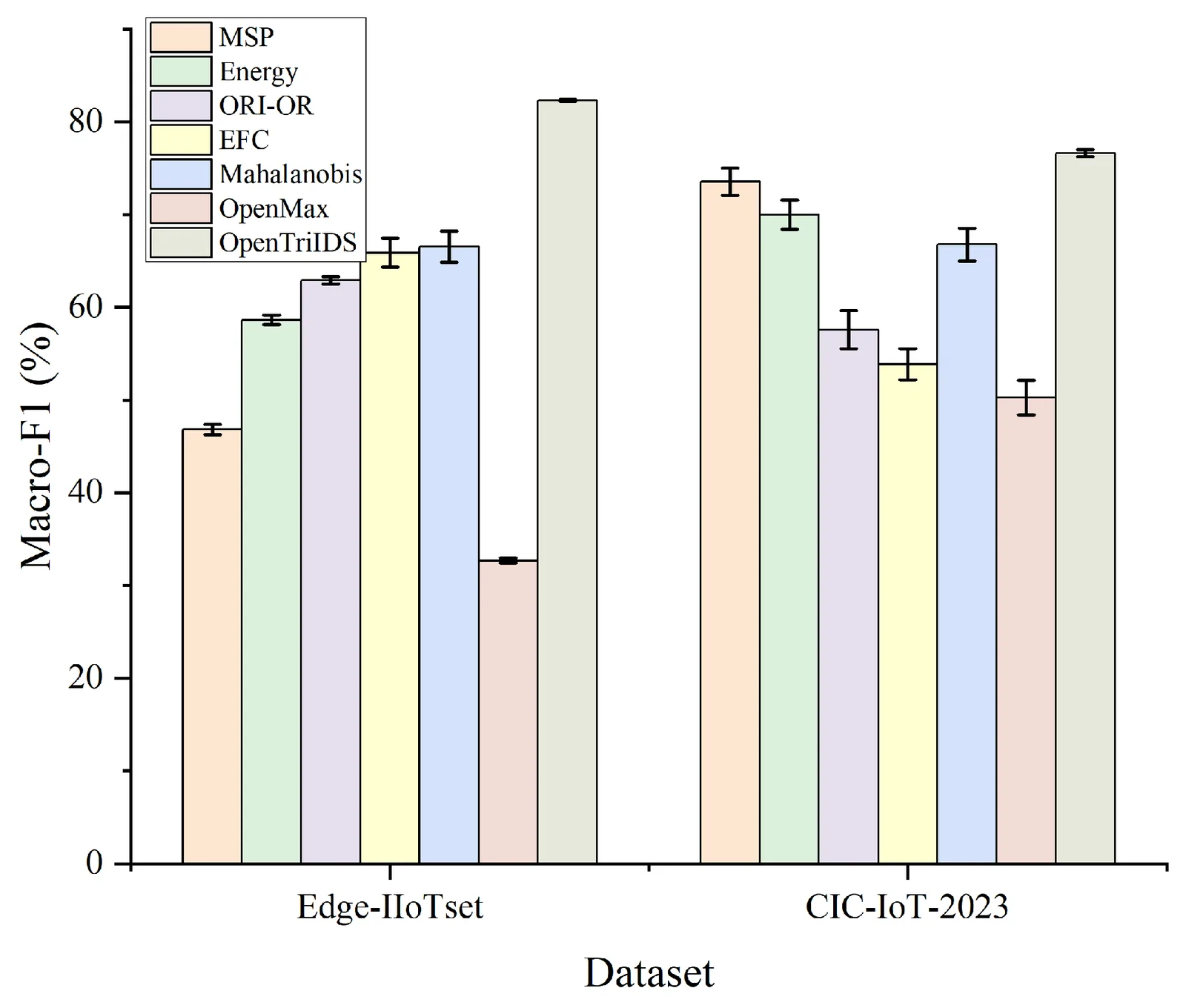
Seven-method S1 macro-F1 for Edge-IIoTset and CIC-IoT-2023.

Figure 10 visualizes the seven-method macro-F1 values in Table 7.

Under these documented operating points, OpenTriIDS achieves the highest macro-F1 among the evaluated methods in both S1 settings. This system-level comparison complements the separate prototype attribution in Table 4; it does not establish superiority at a matched rejection rate or computational budget.

On Edge S1, OpenTriIDS exceeds the next-highest ORI-OR macro-F1 by 5.54 percentage points. On CIC S1, it exceeds the strongest comparator, MSP, by 3.25 percentage points. These values describe the evaluated operating points; they do not establish a universal ranking of unknown recall, false alarms, computational cost, or matched-budget performance.

### 4.5. Sensitivity, Robustness, and Scope

**Figure 11:**
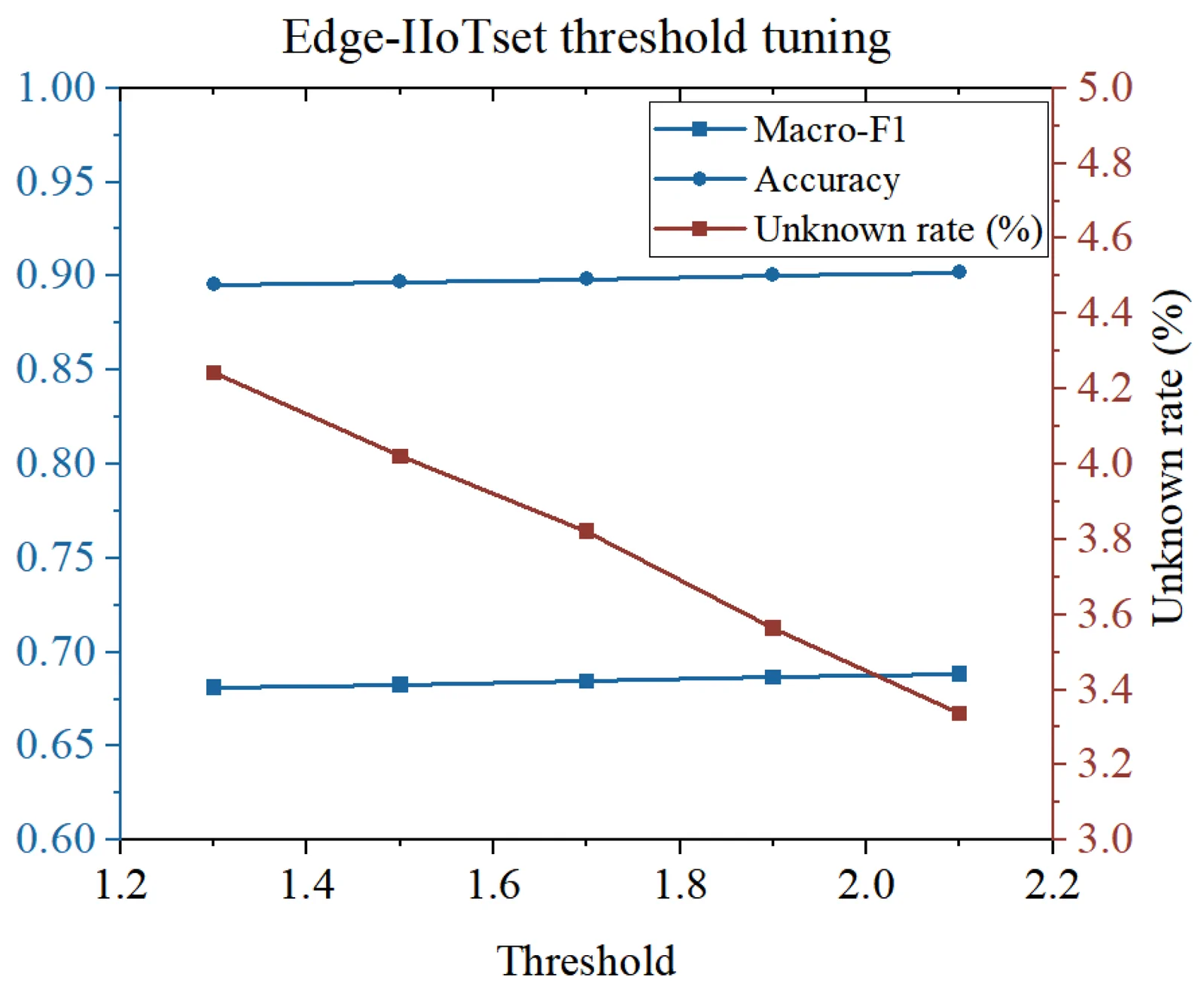
Unknown-distance threshold sensitivity on Edge-IIoTset S1.

**Figure 12:**
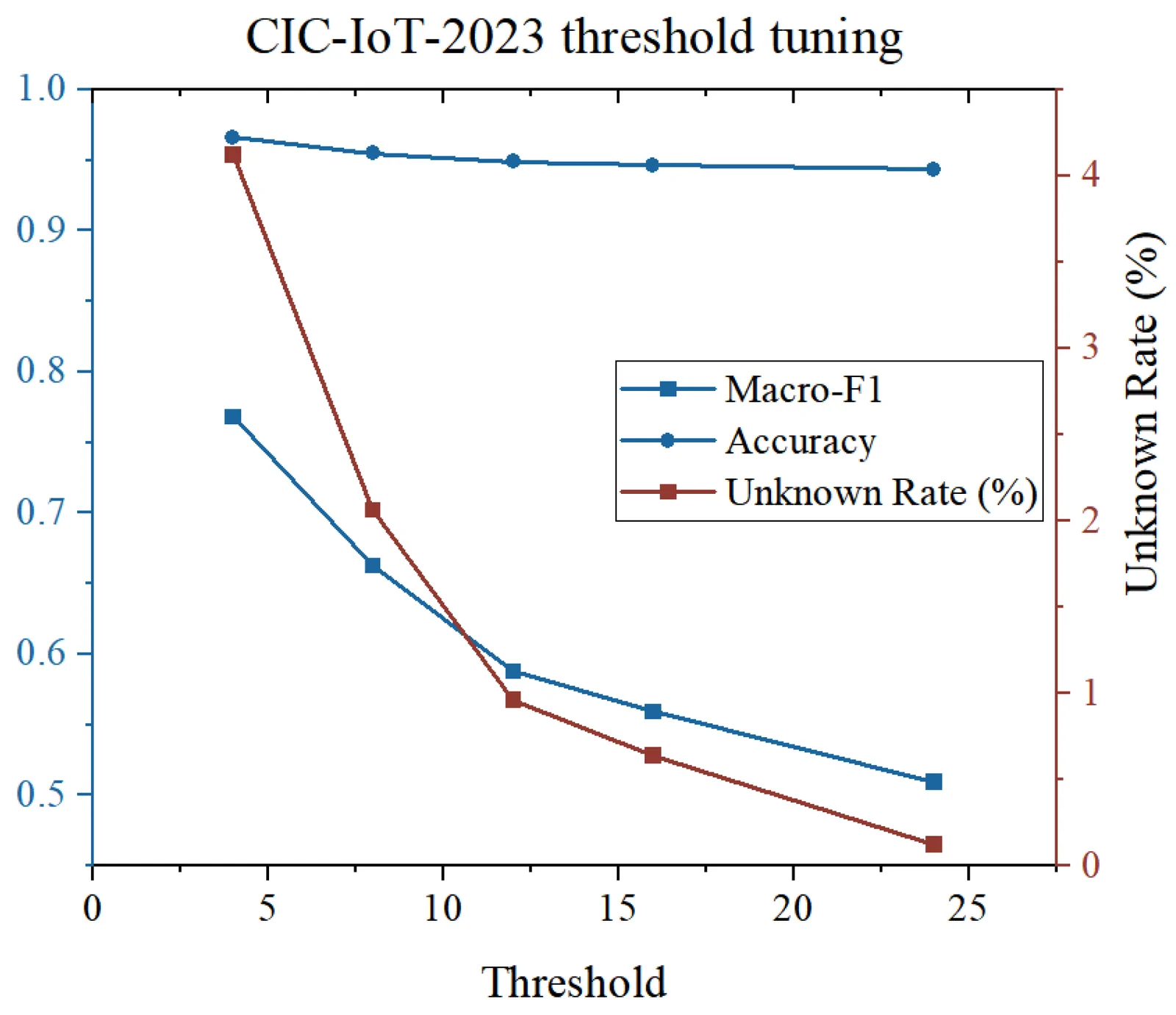
Unknown-distance threshold sensitivity on CIC-IoT-2023 S1.

The representative S1 protocol also examines the sensitivity of the unknown-distance boundary; the next two figures summarize the evaluated sweeps, see Figure 11 and Figure 12.

On Edge-IIoTset, moving $\tau_{u}$ from 1.3 to 2.1 changes macro-F1 from 68.13% to 68.83% and slightly reduces the final unknown-assignment rate. CIC-IoT-2023 exhibits a substantially different distance range, approximately 4–24 in the evaluated sweep; macro-F1 decreases when the boundary is moved too far outward. These results support treating $\tau_{u}$ as a dataset- and protocol-specific operating choice rather than a universal distance scale.

As an additional control, training-stage validation retuning gives $\tau_{u}=2.1$ for Edge-IIoTset and $\tau_{u}=4.0$ for CIC-IoT-2023. This sensitivity control does not use held-out test labels for threshold selection. The qualitative ordering across held-out settings is unchanged under these retuned values; therefore, the representative S1 result is not tied to a single distance boundary. This analysis belongs to the representative protocol; the controlled comparison across held-out-family settings retains the prespecified thresholds 0.75 and 1.7 for paired attribution.

#### Robustness and Scope

All three holdouts and seeds are reported under fixed feature, gate, and distance settings. Seed summaries describe observed partition and model variation within one underlying population and are interpreted as descriptive robustness evidence. One-versus-three-prototype ablations do not establish an optimal count. Other prototype counts and alternative distances remain untested; threshold sensitivity is not included in the current controlled comparison across held-out-family settings.

Routing spans 13.02–35.88% and therefore controls the distance-scored subset, not runtime directly (Section 3). Matched-gate ablations and same-partition-method comparisons answer attribution and predictive-comparison questions, respectively. An accuracy–cost frontier additionally requires a target population, validation-selected operating points, frozen testing, and comparable end-to-end timing. Equal final rejection alone does not equalize secondary computation.

Feature-dependent collision screening changes the evaluated population. Row stratification and exact deduplication leave possible near-duplicate, session, capture, and device dependencies. The evaluation remains limited to two datasets and does not include cross-domain transfer or online updating; these conditions bound generalizability.

## 5. Conclusions

OpenTriIDS distinguishes uncertain predictions from insufficient known-class support by assessing confidence-selected traffic with training-derived prototypes. The representative S1 analysis reports macro-F1 values of 68.44% on Edge-IIoTset and 76.81% on CIC-IoT-2023, with routing rates of 4.53% and 4.13%, respectively. Its ablations show that the confidence gate defines the selected subset, while the three-prototype support rule improves macro-F1 and changes the balance between known retention and unknown recall; the magnitude and direction depend on the dataset and operating point. A complementary three-seed matched-gate analysis reports higher macro-F1 for OpenTriIDS than for confidence-only and centroid rejection in all Edge-IIoTset and CIC-IoT-2023 S1–S3 settings, while the unknown-recall panels show a dataset- and holdout-dependent trade-off. The seven-method S1 comparison reports the highest macro-F1 for OpenTriIDS at the documented operating points, with 68.44% on Edge-IIoTset and 76.81% on CIC-IoT-2023. These findings support conditional known-class recovery under the evaluated protocols, with reduced known false rejection weighed against missed unknown attacks and method-specific precision–recall trade-offs; they do not establish a universal ranking at matched rejection or computational budgets.

These findings are limited to fixed operating points on two screened, row-partitioned benchmarks; session and device dependencies remain possible, and conditional scoring alone does not establish a runtime advantage. The representative analysis includes a distance-threshold sensitivity check, whereas broader threshold and prototype-count sensitivity under the controlled evaluation across held-out-family settings, alternative distances, matched-budget performance, and newer corpora remains for future work. Cross-domain validation requires common feature and label mappings, source-fitted preprocessing, and decision rules to be frozen before target evaluation. Online continual learning could incorporate analyst-confirmed rejected samples into prototype or detector updates, supported by drift monitoring, label-quality controls, replay or regularization against forgetting, and rollback safeguards. These extensions would test the transferability and operational value of the static support mechanism evaluated here.

## Figures and Tables

**Table 1:** Controlled-protocol sample screening. Counts show successive removals from the benchmark-derived inputs.

Quantity	Edge-IIoTset	CIC-IoT-2023
Input rows before screening	157,800	712,311
Invalid rows removed	0	14
Cross-label feature-conflict rows removed	17,799	110,802
Same-label duplicate rows removed	1270	107,367
Retained rows	138,731	494,128
Numerical features	32	39
Retained original classes	15	34

**Table 2:** Representative S1 operating points. Values are percentages from single S1 runs; “–” means not applicable.

Dataset	Method	Accuracy (%)	Macro-F1 (%)	Routing (%)	Unknown Assignment (%)
Edge-IIoTset	RF closed-set baseline	96.84	93.76	–	–
Edge-IIoTset	RF open-set baseline	90.10	60.01	–	–
Edge-IIoTset	RF confidence-only control	90.68	61.14	4.53	4.53
Edge-IIoTset	RF centroid control	90.95	60.90	4.53	–
Edge-IIoTset	OpenTriIDS (RF + explicit rejection)	89.83	68.44	4.53	3.82
Edge-IIoTset	XGBoost + explicit unknown rejection	89.80	62.50	3.76	2.18
Edge-IIoTset	HistGB + explicit unknown rejection	89.33	62.46	5.11	2.84
Edge-IIoTset	MLP + explicit unknown rejection	80.98	47.92	18.45	5.60
CIC-IoT-2023	RF closed-set baseline	99.19	91.07	–	–
CIC-IoT-2023	RF open-set baseline	94.42	52.72	–	–
CIC-IoT-2023	RF confidence-only control	94.32	50.74	4.13	4.13
CIC-IoT-2023	RF centroid control	94.30	50.08	4.13	–
CIC-IoT-2023	OpenTriIDS (RF + explicit rejection)	96.61	76.81	4.13	4.13
CIC-IoT-2023	XGBoost + explicit unknown rejection	96.74	76.27	3.91	3.91
CIC-IoT-2023	HistGB + explicit unknown rejection	96.39	73.59	3.72	3.71
CIC-IoT-2023	MLP + explicit unknown rejection	96.03	70.78	3.55	3.55

**Table 3:** Partition sizes and routing rates (%).

Setting	Train	Validation	Test Known	Test Unknown	Gate Rate
Edge S1	82,514	27,505	27,505	1207	13.02 ± 0.18
Edge S2	71,972	23,991	23,991	18,777	35.88 ± 0.39
Edge S3	71,000	23,667	23,667	20,397	34.28 ± 0.33
CIC S1	292,228	97,410	97,410	7080	30.40 ± 0.22
CIC S2	294,874	98,292	98,292	2670	27.75 ± 0.01
CIC S3	296,307	98,770	98,770	281	27.32 ± 0.18

**Table 4:** Matched-gate module ablation: operational macro-F1 (%).

Setting	Confidence-Only	Centroid	OpenTriIDS
Edge S1	75.73 ± 0.20	77.52 ± 0.09	82.30 ± 0.10
Edge S2	60.59 ± 0.04	78.79 ± 0.45	83.60 ± 0.44
Edge S3	67.50 ± 1.32	78.60 ± 0.18	85.45 ± 0.25
CIC S1	60.43 ± 0.22	72.63 ± 0.33	76.62 ± 0.37
CIC S2	56.16 ± 0.25	65.22 ± 0.43	68.82 ± 0.44
CIC S3	53.17 ± 0.22	58.00 ± 0.26	59.44 ± 0.27

**Table 5:** Unknown handling and known false rejection (%).

Setting	Variant	Unknown Recall	Unknown Precision	Known FR	Unknown Assignment
Edge S1	Confidence-only	74.29 ± 0.96	23.99 ± 0.36	10.33 ± 0.17	13.02 ± 0.18
Edge S1	Centroid	74.29 ± 0.96	27.75 ± 0.14	8.49 ± 0.10	11.25 ± 0.13
Edge S1	OpenTriIDS	52.64 ± 0.62	51.07 ± 0.23	2.21 ± 0.04	4.33 ± 0.07
Edge S2	Confidence-only	67.06 ± 1.09	82.05 ± 0.45	11.48 ± 0.17	35.88 ± 0.39
Edge S2	Centroid	51.37 ± 1.13	81.45 ± 0.49	9.15 ± 0.14	27.69 ± 0.46
Edge S2	OpenTriIDS	8.71 ± 0.15	80.97 ± 3.18	1.61 ± 0.36	4.73 ± 0.26
Edge S3	Confidence-only	67.04 ± 0.75	90.53 ± 0.14	6.04 ± 0.03	34.28 ± 0.33
Edge S3	Centroid	48.17 ± 0.47	89.15 ± 0.14	5.05 ± 0.04	25.01 ± 0.21
Edge S3	OpenTriIDS	21.63 ± 2.43	94.25 ± 0.93	1.13 ± 0.15	10.62 ± 1.13
CIC S1	Confidence-only	83.68 ± 1.13	18.65 ± 0.13	26.53 ± 0.17	30.40 ± 0.22
CIC S1	Centroid	83.68 ± 1.13	41.92 ± 0.26	8.43 ± 0.03	13.53 ± 0.10
CIC S1	OpenTriIDS	82.73 ± 1.48	54.84 ± 0.19	4.95 ± 0.06	10.22 ± 0.16
CIC S2	Confidence-only	66.59 ± 2.97	6.35 ± 0.28	26.69 ± 0.07	27.75 ± 0.01
CIC S2	Centroid	66.59 ± 2.97	17.35 ± 0.67	8.62 ± 0.06	10.15 ± 0.08
CIC S2	OpenTriIDS	66.18 ± 3.11	25.25 ± 0.76	5.32 ± 0.17	6.93 ± 0.22
CIC S3	Confidence-only	87.19 ± 1.23	0.91 ± 0.01	27.15 ± 0.18	27.32 ± 0.18
CIC S3	Centroid	87.19 ± 1.23	2.62 ± 0.01	9.24 ± 0.11	9.46 ± 0.11
CIC S3	OpenTriIDS	86.95 ± 0.82	3.98 ± 0.03	5.97 ± 0.07	6.20 ± 0.08

**Table 6:** Normal-traffic and known-attack errors (%).

Setting	Normal False Alarm	Confidence-Only Attack FR	Centroid Attack FR	OpenTriIDS Attack FR
Edge S1	3.61 ± 0.04	11.78 ± 0.22	9.55 ± 0.13	2.52 ± 0.04
Edge S2	3.35 ± 0.07	13.54 ± 0.21	10.64 ± 0.16	1.88 ± 0.45
Edge S3	3.47 ± 0.17	6.76 ± 0.06	5.54 ± 0.10	1.27 ± 0.15
CIC S1	31.89 ± 0.71	26.36 ± 0.18	7.62 ± 0.05	4.05 ± 0.09
CIC S2	32.23 ± 0.30	26.53 ± 0.07	7.82 ± 0.05	4.43 ± 0.17
CIC S3	38.12 ± 0.84	26.79 ± 0.21	8.25 ± 0.13	4.88 ± 0.10

**Table 7:** Seven-method S1 macro-F1 comparison (%).

Dataset	MSP	Energy OOD	ORI-OR	EFC	Mahalanobis	OpenMax	OpenTriIDS
Edge-IIoTset	46.82 ± 0.57	48.67 ± 0.37	62.90 ± 0.39	55.88 ± 1.57	39.93 ± 1.68	32.69 ± 0.30	68.44 ± 0.10
CIC-IoT-2023	73.56 ± 1.48	55.95 ± 1.56	57.57 ± 2.07	53.85 ± 1.69	68.55 ± 1.76	50.27 ± 1.85	76.81 ± 0.37

## Data Availability

Edge-IIoTset and CIC-IoT-2023 are public benchmark datasets. The implementation, feature definitions, split records, and processed experimental materials are available from the corresponding authors upon reasonable request, subject to the source datasets’ terms of use. The code and processed materials are not currently deposited in a public repository.
